# Hidden Cobalt Hazards in Children: A Soil Risk Assessment at the Sokolov–Sarbai Complex

**DOI:** 10.3390/toxics14070617

**Published:** 2026-07-15

**Authors:** Bekmyrza Zhumash, Yskak Aliya, Paramonova Tatyana, Irzhanov Zhassulan, Nugmanov Almabek, Yermoldina Gulnaz, Tokusheva Assel, Fominov Vladimir, Bulaev Aleksandr, Lyanga Petr, Zhumalynov Kuanysh, Bozhekenova Zheniskul

**Affiliations:** 1 Research Institute of Innovational Technogies, Akhmet Baitursynuly Kostanay Regional University, 28/1 Abai Str., Kostanay 110000, Kazakhstan; 5112431@mail.ru (B.Z.); alia-almaz@mail.ru (Y.A.); tapara@mail.ru (P.T.); almabek.nugmanov@bk.ru (N.A.); gulnazyermoldina@gmail.com (Y.G.); assel.salimzhan@gmail.com (T.A.); vladimirfominov49eng@gmail.com (F.V.); sadhgy19@gmail.com (L.P.); zhumalynov.k@mail.ru (Z.K.); bozhekenova@mail.ru (B.Z.); 2 Research Center of Biotechnology, Russian Academy of Sciences, 7/2 60-Letiya Oktyabrya Ave., 117312 Moscow, Russia; bulaev.inmi@yandex.ru

**Keywords:** health risk assessment, hazard index, cobalt, oral bioaccessibility, reference dose uncertainty, children, integrated pollution indices, iron ore mining

## Abstract

Iron ore mining is often assumed to pose a low soil-contamination risk because magnetite is dense and metal-poor. We tested this assumption around the Sokolov–Sarbai magnetite–skarn complex (Kostanay Region, Kazakhstan) by sampling soils at four settlements and a nearby background site, analyzing sixteen elements in pseudo-total and exchangeable forms, and comparing classical pollution indices with disaggregated US EPA health risk metrics on the same dataset. The two frameworks diverge sharply. Integrated indices (PLI, PERI) classify the area as low-risk, yet the child hazard index (US EPA cobalt reference dose) exceeds one at all four settlements, driven almost entirely by cobalt—a single, highly mobile element released from the late-stage sulfide paragenesis of the host skarn against an already cobalt-rich regional background. This relative result—cobalt as the dominant contributor and Rudny City as the most affected settlement—is robust to exposure-parameter variation and, at Rudny City and Konstantinovka, to unfavorable bioaccessibility assumptions. Carcinogenic risk stays within the acceptable range and reflects regional-background nickel and chromium rather than a mining increment. For single-element, regionally elevated contaminants, integrated indices can therefore understate a genuine child health hazard; adequate assessment requires mobile-fraction characterization and bioaccessibility-explicit risk computation.

## 1. Introduction

Mining-impacted soils around iron ore operations have been studied less intensively than those around sulfide-ore (Cu, Pb, Zn) or coal-fly-ash districts [1], despite the large physical footprint of iron ore production [2]. Magnetite-dominated ore is conventionally assumed to pose a low ecological risk [3] because iron has high regulatory thresholds and the dominant ore mineral is dense and poorly wind-transported. This view assumes that mine-waste chemistry mirrors the bulk ore, and it ignores the different mineralogy of waste rock, low-grade material and tailings, where late-stage sulfide and silicate phases concentrate trace metals that are virtually absent from primary magnetite.

Cobalt is the trace element of principal concern in this setting. It is an established human toxicant—chronic oral exposure has been linked to thyroid, cardiac and hematological effects [4,5,6,7]—and young children are especially vulnerable because they ingest soil and dust at far higher body weight-normalized rates than adults [8,9]. Elevated trace-element burdens have been documented even in urban playground soils that children directly contact [10]. Because incidental soil ingestion is the dominant residential exposure route for non-volatile metals [9], a soil compartment enriched in cobalt and, critically, in its mobile and bioaccessible fraction translates directly into elevated child health risk. The concern is not confined to a single deposit: magnetite–skarn and related iron ore systems are distributed worldwide, with documented Co-bearing examples in China, Iran, the Philippines, the United States and Kazakhstan [11,12,13], so a cobalt-driven soil hazard that conventional screening overlooks would be a generic and largely unrecognized feature of a widespread class of mining operations.

The assessment tools themselves are part of the problem. The integrated pollution indices that dominate the mining-impact literature—the pollution load index, the geoaccumulation index and the potential ecological risk index [14,15,16,17]—aggregate many elements against a regional baseline and are, by construction, insensitive to a single, strongly enriched element diluted among unenriched ones. Where the regional background is itself elevated over an ore-bearing formation, that enrichment is already absorbed into the index denominator, so the single-element anomaly is masked. Disaggregated, receptor-specific human-health risk assessment following the US EPA framework [18] can in principle recover a single-element hazard that these indices miss, yet the two approaches are rarely applied side by side to the same iron ore-impacted dataset.

Iron skarns are a relevant case. Their late-stage paragenesis includes sulfide and, where arsenic is present, sulfarsenide phases (pyrite, pyrrhotite, chalcopyrite, sphalerite, arsenopyrite, cobaltite) that concentrate cobalt, whereas the magnetite ore itself is a poor host—reported Co reaches tens of thousands of ppm in sulfarsenides against single-digit ppm in magnetite ([11,12]; Section 4.1). The Sokolov–Sarbai deposits of the Turgai belt (Kazakhstan), part of the Valerianovka arc on the eastern margin of the South Urals [19], show scapolite–(albite)–magnetite alteration with documented late-stage sulfide mineralization. With over 3 Gt of cumulative iron ore resource, the complex has been mined since 1958 and dominates the economy of Rudny City (population 123,240 as of 1 July 2024).

The present study therefore asks three questions. First, whether iron ore mining around the Sokolov–Sarbai complex produces a measurable, health-relevant trace-element signal in the soils of the surrounding settlements. Second, whether such a signal would be captured by the conventional integrated pollution indices that dominate the mining impact literature, or whether it would be masked by them. Third, which contaminant and which exposure pathway, if any, should be the public-health priority for the residents of Rudny City and the neighboring settlements.

## 2. Materials and Methods

### 2.1. Study Area

**Figure 1:**
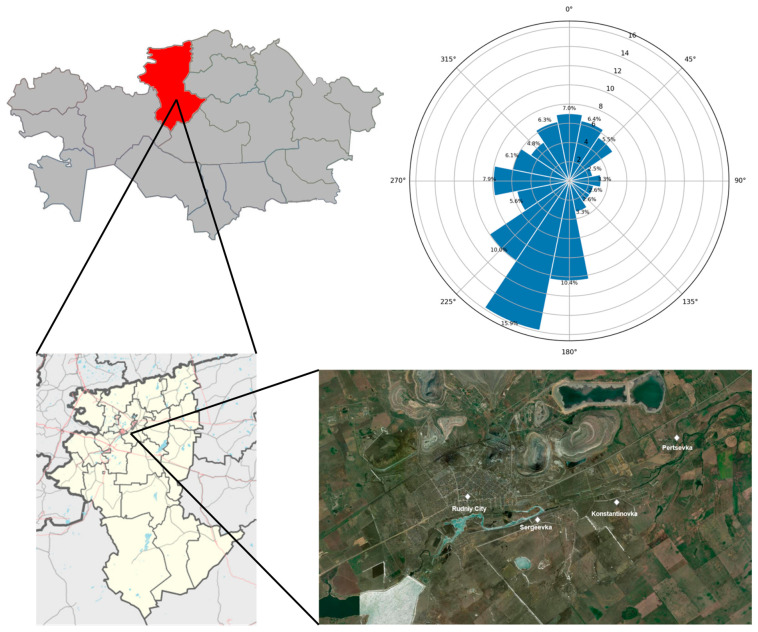
Location of the study area and sampling design. Four settlements around the Sokolov–Sarbai iron ore complex (Rudny City, Sergeevka, Konstantinovka and Pertsevka; Diamond-shaped square). The wind rose (inset, top right) shows the frequency distribution of wind direction (16 sectors, % of annual hours from the indicated bearing; Rudny City station, 2016–2026). A background reference site was located ~50 km west of the mining footprint on geomorphologically similar steppe.

The Sokolov–Sarbai Mining and Processing Association (SSGPO; Eurasian Resources Group) operates four open-pit mines (Sokolovsky, Sarbaisky, Kacharsky, Korzhinkolskoye) and one underground mine in the Kostanay Region of northern Kazakhstan, processing ~40 Mt of magnetite-rich ore per year. The orebodies are limestone-replacement Fe skarns of the Valerianovka arc (Carboniferous; 336 ± 1 Ma [19]), with scapolite–(albite)–magnetite alteration and late-stage sulfide/sulfarsenide mineralization. The climate is sharply continental (Dfb; mean January and July temperatures −15.6 °C and +21 °C). Prevailing winds are S–SSW (15.9% of annual hours from 180°, 10.4% from 202.5°; Figure 1), i.e., blowing towards the N–NNE, while reverse N–NNE winds carry mine-derived dust back towards the settlements with a combined frequency of ~24%.

Four settlements were studied (Figure 1): Rudny City (123,240 inhabitants, ~4.5 km SSW of open-pit OP1), Sergeevka (~3.3 km S of OP2), Konstantinovka (~4.4 km S of waste-dump WD2.1) and Pertsevka (~2.4 km SE of the tailings storage facility TSF), with a background site ~50 km west on geomorphologically similar steppe. At each impacted settlement, three soil pits were established within a ~3 m radius at a single representative location in or near the residential area. At the background site, three composite samples per horizon were collected, each pooling nine subsamples within a 50 m radius. Every pit (or composite) was sampled at three depth horizons (0–10, 10–20, 20–30 cm), giving *n* = 9 per site (*n* = 45 pseudo-total and *n* = 45 exchangeable across all five sites); the complete per-sample dataset (pseudo-total, mobile and physico-chemical) is compiled in Table S1. Co-located herbaceous biomass was also collected but is treated as a separate compartment outside the scope of this soil assessment (Section 4.4).

### 2.2. Analytical Procedure

Air-dried soils (7–10 days, room temperature) were sieved to <2 mm and ground in an agate mill to <0.1 mm. Pseudo-total concentrations of 16 elements (Al, Ca, Cd, Co, Cr, Cu, Fe, K, Mg, Mn, Na, Ni, P, Pb, S, Zn) were determined by microwave-assisted acid digestion (US EPA Method 3051A): 0.5000 g of soil was digested with 9 mL HNO_3_ (65%) and 3 mL HCl (37%) in a Milestone ETHOS EASY microwave system (Milestone Srl, Sorisole, Italy) at 175 °C; digests were then filtered through ashless quantitative filter paper (Blue Ribbon grade; Ø 15.0 cm; residue on ignition ≤ 0.00156 g; Melior XXI LLC, Moscow, Russia) and made up to 100 mL with deionized water. Mobile (exchangeable) fractions of the same elements were extracted with neutral 1 M ammonium acetate (CH_3_COONH_4_, pH 7.0; soil:solution ratio 1:10; 2 h on a horizontal shaker) and filtered through the same ashless paper.

All digests and extracts were analyzed on a Thermo Scientific iCAP PRO XP Duo ICP-OES (Thermo Fisher Scientific, Bremen, Germany) operated in dual (axial and radial) viewing mode. Full instrument operating conditions, spectral-line selection, calibration linearity (R^2^), precision (RSD) and detection/quantification limits are given in Table S2 [20]. Quantification used external calibration with a blank plus three non-zero levels (0.1, 1 and 5 mg L^−1^ for the elements in the higher-range standard mix and 0.01, 0.1 and 1 mg L^−1^ for Pb) prepared from multi-element standards (NPP Skat, Novosibirsk, Russia) and rebuilt at the start of each analytical session. Each solution was measured in duplicate and reported as the mean. In parallel, water-suspension pH and total dissolved solids (MARK-901 pH-meter and MARK-603 conductivity meter, Vzor LLC, Nizhny Novgorod, Russia), humus (Tyurin method), particle-size distribution, exchangeable bases (CEC), water-extract mineralization and moisture were determined for each sample (Table S3).

Quality control comprised calibration linearity, internal-standard monitoring and replicate precision. Instrumental drift and matrix effects were corrected on-line with a scandium internal standard (10 mg L^−1^) introduced at equal concentration to every blank, standard and sample; its recovery remained within 90–110% throughout each run. Instrumental limits of detection were derived from the calibration blank as 3σ, with LOQ = 3.3 × LOD (10σ), and solution limits were converted to a sample basis using the respective dilution factors. No certified reference material, procedural blank or matrix-spike recovery test was included in this campaign. Analytical trueness was therefore constrained by calibration and internal-standard drift correction rather than validated against a matrix-matched certified reference, which is recorded as a limitation (Section 4.4). Accordingly, the reported concentrations and the derived indices are treated as relative, internally consistent measures rather than reference-traceable values. Because the principal conclusions of this study are relative—the dominance of cobalt within the child hazard index and the rank order of the sites—they are robust to any systematic recovery offset, which would shift all sites in the same direction without altering their relative structure.

### 2.3. Geochemical Indices, Mobility and Reference Baselines

Two aqua-regia-compatible databases were used to judge the absolute magnitude of the measured concentrations: the FOREGS Geochemical Atlas of Europe subsoil aqua-regia medians ([21]; Co median 8.0 mg kg^−1^) and the GEMAS agricultural/grazing-land subsoil aqua-regia medians ([22]; Co median 7.78 mg kg^−1^). The Upper Continental Crust (UCC [23]) and world soil mean ([24]) are reported for context only, with the caveat that these are true-total digestions and our aqua-regia values understate true totals by 15–30% for Co in silicate-bearing matrices, so UCC and world-soil multipliers are lower bounds of true exceedance. These geochemical benchmarks are distinct from the analytical certified reference material (none was used; Section 2.2) and from the local background concentrations that serve as the divisor in the enrichment indices below.

The contamination factor (CF), geoaccumulation index (Igeo), pollution load index (PLI) and potential ecological risk index (PERI) were calculated for the priority trace elements as follows:

$$CF=\frac{C_{\mathrm{sample}}}{C_{\mathrm{background}}}\;$$

$$I_{\mathrm{geo}}={log}_{2}\left(\frac{C_{\mathrm{sample}}}{1.5\cdot C_{\mathrm{background}}}\right)$$

$$PLI={\left({CF}_{1}\cdot {CF}_{2}\cdots {CF}_{n}\right)}^{1/n}$$

$$E_{r}=T_{r}\cdot CF$$

$$PERI=\sum E_{r}$$

where *C_sample_* is the element concentration in the sample, *C_background_* the median concentration at the unimpacted local background site, *Tr* the element-specific toxicity-response coefficient (Cd 30; Co 5; Cr 2; Cu 5; Ni 5; Pb 5; Zn 1; Mn 1), and *n* the number of elements. Referencing the indices to the already cobalt-rich local background is the conservative choice for the index–risk comparison, since it yields lower index values than a global baseline. The Hakanson threshold for “low ecological risk” is *PERI* < 150. A more conservative threshold of 110 [25] is reported as a secondary check, and the two yield qualitatively identical conclusions here.

The mobility factor (*MF*) quantifies the readily extractable fraction of each element:

$$MF=\frac{C_{\mathrm{mobile}}}{C_{\mathrm{pseudo-total}}}\times 100\%$$

where *C_mobile_* is the ammonium-acetate-extractable concentration and *C_total_* the pseudo-total (aqua-regia-extractable) concentration. *MF* is an operationally defined metric of the readily extractable fraction [26,27,28], not a direct measure of in vivo bioavailability or in vitro bioaccessibility (UBM/SBET type assays). Bioavailability-relevant risk is handled separately through the literature-based bioaccessibility correction (Section 2.5). Additional geochemical indices (enrichment factor, modified degree of contamination, Nemerow index) and vertical-profile analyses are reported in the Supplementary Information.

### 2.4. Multivariate Source Attribution

Principal component analysis (PCA) was performed on log_10_-transformed and Z-standardized pseudo-total concentrations at the sample level (16 elements, *n* = 45). The number of components to retain was set by Horn’s parallel analysis [29], and the retained loadings were varimax-rotated [30,31]. Sampling adequacy was assessed by the Kaiser–Meyer–Olkin measure and Bartlett’s test of sphericity [32]. A parallel 22-variable model incorporating six physico-chemical covariates was run as a robustness check and reproduced the same factor structure; its full loadings, communalities and rotation diagnostics are given in the Supplementary Information (Table S4). For settlement-level clustering, agglomerative hierarchical cluster analysis (Ward.D2 linkage) was performed on Z-standardized site-mean profiles, and cluster stability was verified by independent k-means clustering.

### 2.5. Health Risk Assessment and Uncertainty

The non-carcinogenic hazard index (HI) and total carcinogenic risk (*TCR*) were calculated following US EPA RAGS Part E [18] for adult (*BW* = 70 kg, *ED* = 24 yr, IngR = 100 mg d^−1^) and child (*BW* = 15 kg, *ED* = 6 yr, IngR = 200 mg d^−1^; *EF* = 350 d yr^−1^ for both) receptors across three exposure pathways—incidental oral ingestion, dermal contact and inhalation:

$${ADD}_{\mathrm{ing}}=\frac{C\cdot IngR\cdot EF\cdot ED\cdot CF_{u}}{BW\cdot AT}$$

$${ADD}_{\mathrm{derm}}=\frac{C\cdot SA\cdot AF\cdot ABS\cdot EF\cdot ED\cdot CF_{u}}{BW\cdot AT}$$

$${ADD}_{\mathrm{inh}}=\frac{C\cdot InhR\cdot EF\cdot ED}{PEF\cdot BW\cdot AT}$$

$${HQ}_{i}=\frac{{ADD}_{i}}{{RfD}_{i}}$$

$$HI=\sum {HQ}_{i}$$

$${CR}_{i}={ADD}_{i}\cdot {SF}_{i}$$

$$TCR=\sum {CR}_{i}$$

$${ADD}_{\mathrm{ing},\mathrm{bioacc}}={ADD}_{\mathrm{ing}}\cdot BAF$$

where *C* is the soil concentration; the remaining symbols are the standard RAGS Part E exposure parameters, and *RfDi* and *SFi* the reference dose and oral slope factor of element *i* (all input values in (Table S5). Non-carcinogenic reference doses were taken from US EPA IRIS where available. Cobalt has no oral IRIS RfD; the US EPA PPRTV chronic provisional oral RfD of 3 × 10^−4^ mg kg^−1^ d^−1^ (EPA/690/R-08/008F), carried in the current EPA Regional Screening Level tables, was adopted as the sole cobalt reference dose. Oral slope factors, not provided in IRIS for Cd, Pb, Cr or Ni, were taken as provisional values consistent with the California EPA OEHHA Toxicity Criteria Database [33]; the Cr value assumes a 10% Cr(VI) fraction, which is conservative for these slightly alkaline soils. The provisional, non-IRIS nature of these slope factors is a limitation common to all RAGS Part E studies for these elements.

Direct in vitro bioaccessibility (UBM/SBET, ISO 17924 [34,35]) was not performed. Literature-derived bioaccessibility factors (*BAF*) were applied to the oral ingestion pathway only [36], centered on published soil-matrix medians: Co *BAF* = 0.20 (range 0.10–0.40) [37], extrapolated from in vitro measurements on urban, smelter- and grinding-impacted soils and dusts [38,39,40,41], none of which were a magnetite–skarn product, with analogous element-specific values for Pb, Cd, Cr, Ni, Cu, Zn and Mn (Table S5) [42]. No direct measurement of Co bioaccessibility in iron-skarn soil has been published, and the resulting toxicity-value and bioaccessibility uncertainties are discussed in Section 4.4. Uncertainty was propagated in three layers, reported together in Section 3.5: a deterministic one-at-a-time (OAT) sensitivity analysis of the exposure parameters (*IR* ±30%, *EF* ±30%, *BW* ±15%, *ED* ±30%); two combined optimistic/pessimistic exposure scenarios (HI multipliers 0.43 and 1.99); and a stochastic Monte Carlo simulation (*n* = 10,000) treating each element’s *BAF* as a lognormal variable (σ_log = 0.4–0.5, clipped to [0.01, 1.0]). Per-layer numerical detail is provided in Tables S6–S9.

### 2.6. Statistical Analysis

Descriptive statistics were computed per site, per horizon and per site × depth. Normality was assessed by the Shapiro–Wilk test on raw and log_10_-transformed data, and homogeneity of variances by Levene’s and Bartlett’s tests (Table S10). Associations between mobile concentrations and soil physico-chemical properties are reported as Spearman’s ρ with the Benjamini–Hochberg false discovery rate procedure (Table S11). Site- and depth-level differences were examined with linear mixed-effects models, which reduced to a two-way ANOVA where the random-effect variance was singular under the available replication (Table S10). All analyses used Python 3.12.10 with NumPy 2.1.3, SciPy 1.14.1, statsmodels 0.14.4, pingouin 0.5.5, scikit-learn 1.5.2 and factor-analyzer 0.5.1.

## 3. Results

### 3.1. Element Concentrations

**Figure 2:**
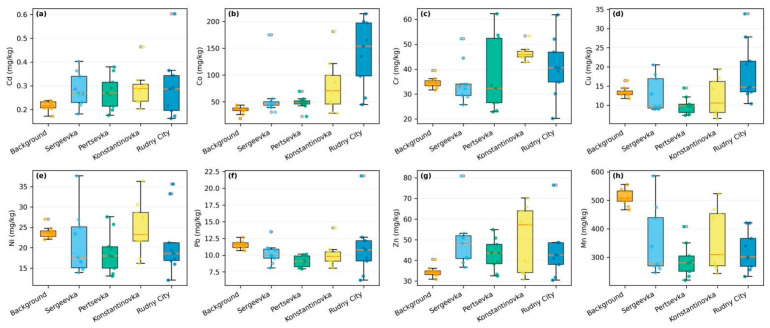
Site-level distribution of pseudo-total (aqua-regia-extractable) concentrations of eight priority trace metals in topsoil. Panels (**a**–**h**): Cd, Co, Cr, Cu, Ni, Pb, Zn and Mn (mg kg^−1^ dry weight). Boxes show the interquartile range and median; whiskers extend to 1.5 × IQR; outliers are shown as open circles, individual samples as filled dots. Sites: background; Sergeevka; Pertsevka; Konstantinovka; Rudny City. *n* = 9 per site (3 points × 3 horizons).

The first question—is there a measurable, health-relevant signal?—is answered directly by the concentrations: of the sixteen elements, only cobalt deviates sharply from regional and global baselines (Figure 2; Table S11). The median Co concentration in Rudny City soil (154 mg kg^−1^, site median) exceeds the FOREGS pan-European subsoil aqua-regia baseline (8.0 mg kg^−1^) by 19.2× and the GEMAS baseline (7.78 mg kg^−1^) by 19.8×, with individual maxima at 27× and 28× (Table 1). Even the background site (35 mg kg^−1^) is 4.4× the European baseline, reflecting an elevated regional lithochemistry over the host formation. No other element is comparably enriched: median Cd, Cr, Ni, Pb and Zn are 0.7–2.5× FOREGS and Fe is 0.8–1.2× FOREGS (complete per-element ratios in Table S13).

Concentrations were measured by US EPA Method 3051A (microwave-assisted HCl + HNO_3_ digestion), which is operationally equivalent to aqua regia for chalcophile elements (recoveries within ±10–15% [43,44,45]), so the FOREGS and GEMAS subsoil aqua-regia medians are directly comparable; UCC ratios are a conservative lower bound. Iron shows no detectable surface enrichment despite the proximity of a 3 Gt orebody, for two reasons: aqua regia incompletely recovers silicate-bound Fe, and the high density of magnetite (~5.2 vs. ~2.7 g cm^−3^ for aluminosilicates) limits aeolian transport, favoring proximal deposition over dispersal to settlements 2.4–4.5 km away. This Co–Fe decoupling—Co at 19× baseline, Fe at baseline—is the key geochemical signature separating weathering of the late-stage sulfide assemblage from that of the bulk magnetite ore. Vertical profiles across the three horizons are flat or weakly varying for most elements. Cobalt increases moderately with depth at Rudny City (130 → 165 mg kg^−1^) and weakly at Konstantinovka (Figure S1), but the profiles are reported descriptively only, since several processes could produce them and the surface-horizon concentration and mobility, not the profile shape, govern soil ingestion.

### 3.2. Cobalt Mobility

The signal is not confined to bulk concentration. Cobalt is also the most mobile of the elements determined, which sets the size of the labile pool available for incidental ingestion. Mobility factors are reported as site medians, since the per-sample *MF* is a ratio of two independently determined quantities and its mean is unstable when the denominator is small. Cobalt has by far the highest ammonium acetate-extractable fraction of the elements analyzed (Figure S2): the median *MF* for Co is 68% at Rudny City (87% in the 0–10 cm topsoil), 33–54% in the other settlements and 28% at the background site. In absolute terms, the median mobile Co is 94.5 mg kg^−1^ at Rudny City against 10.0 mg kg^−1^ at the background—a 9.4-fold contrast, compared to a 4.4-fold contrast in the pseudo-total. Other elements with a substantial labile pool are Ca (median *MF* 29–67%), Mn (24–68%) and Cd (15–24%), whereas the sulfide-associated Cu is sparingly mobile (0.1–1.6%) and Fe and Al are essentially immobile (≤2%).

Four of the 45 samples (two at Pertsevka, two at Rudny City) return an MF_Co above 100%. This is not a physically impossible result: the denominator is the pseudo-total (aqua-regia-extractable) concentration, which under-recovers silicate-bound Co, so MF as defined here is not a true sub-fraction ratio. Empirically, all four combine an anomalously low pseudo-total (23–57 mg kg^−1^ against site medians of 48–154 mg kg^−1^) with an at-or-above-median mobile value at the same point, i.e., the two aliquots—one digested, one extracted—disagree. Within-site coefficients of variation for pseudo-total Co reach 22–75% across the five sites, consistent with between-aliquot heterogeneity of the Co-bearing material rather than with a systematic extraction artefact. The same phenomenon was observed at this complex in [46], where such samples were excluded. Given the smaller sample here, all four were retained; the site medians reported above are insensitive to them. *MF* is therefore read as a comparative inter-site indicator of labile-pool enrichment, not as an absolute bioavailable fraction. Exposure-relevant bioavailability is handled separately through the much lower literature *BAF* for Co (Section 3.5), and the risk calculation uses pseudo-total concentrations, so no result in Section 3.5 depends on the *MF* metric.

Set against the national soil-quality standard, this labile pool is diagnostic. The Kazakhstan hygienic norms define a maximum permissible concentration (MPC) for the mobile form of cobalt of 5.0 mg kg^−1^ but set no gross-content MPC for cobalt, so monitoring based on total concentrations alone carries no cobalt trigger. Measured against the mobile-form MPC, cobalt exceeds its standard at every site, from 2.0 × MPC at the background to 18.9 × MPC at Rudny City, whereas the median mobile forms of Ni, Cr, Cu, Zn and Pb remain below their respective MPCs at all sites (0.00–0.62 × MPC), with a single Konstantinovka nickel sample marginally above (1.35 × MPC). Applied to the mobile fraction, the national standard therefore flags cobalt, and only cobalt, and does so even at the regional background, consistent with the elevated regional lithochemistry. One caveat attends the comparison: the mobile-form MPC is operationally defined for the ammonium-acetate buffer at pH 4.8, whereas the present extraction used the neutral (pH 7.0) reagent at the same soil:solution ratio. Because the pH 4.8 buffer is the more aggressive of the two, the values reported here are a conservative estimate of the MPC-comparable pool.

Among the soil physico-chemical properties, mobile Co is most consistently related to soil organic matter: the association is negative (Spearman ρ = −0.51, *p* < 0.001, *n* = 45), it survives control for pH (partial ρ = −0.48), it holds within the impacted settlements alone (ρ = −0.47, *n* = 36), and it retains its sign and significance under leave-one-site-out resampling (Table S11; Figure S3). Mobile Co also co-varies with pH, total mineralization and exchangeable carbonate content, but these associations are between-site contrasts carried by single locations. The pH association vanishes when the background site is excluded (ρ = −0.03, *n* = 36), and the carbonate association when Rudny City is excluded (ρ = +0.01). With four independent impacted locations, they are not interpreted here (Section 4.1).

### 3.3. Integrated Pollution Indices

The second question—would conventional indices register this signal?—is answered in the negative. Despite the pronounced Co signal, the integrated pollution indices fall in low-to-moderate ranges (Table S14). Site-mean *PLI* spans 0.85 (Pertsevka) to 1.34 (Rudny City, 20–30 cm horizon), near the baseline threshold of 1, and site-mean *PERI* ranges 52–99, well below the Hakanson [16] “moderate ecological risk” threshold of 150 (Table S14). The *I_geo_* values bear this out: mean *I_geo_* for Co at Rudny City reaches 1.30, the only element–site combination classified as “moderately polluted” on the Müller scale, and the only one with mean *CF* > 3, with all other elements below 1. This is the documented limitation of integrated indices—a single-element enrichment is masked by dilution against the unpolluted background—and the effect is developed as the central finding of this paper in Section 4.2. Full *CF* and *I_geo_* matrices by element and site are provided in the Supplementary Information.

### 3.4. Multivariate Source Identification

Turning to the source of the signal, the analysis separates cobalt from the host-rock matrix and ties it to a sulfide–Na–P association. Principal component analysis of the 16 pseudo-total concentrations retained three components (Horn’s parallel analysis), explaining 78.3% of variance (unrotated: PC1 47.1%, PC2 22.9%, PC3 8.3%; rotated shares in Table 2). Sampling adequacy was satisfactory (KMO = 0.70), and the diagnostic element Co had communality h^2^ = 0.56. After varimax rotation (Table 2), three interpretable factors of comparable variance emerge.

RC1 (28.3%) loads heavily on Cr, K, Fe, Mg, Al and Ni—the silicate-carbonate matrix of host-rock-derived soil—and represents the host-rock matrix factor; cobalt is virtually absent from it (loading = −0.10), so Co variation is structurally independent of the lithogenic matrix. RC2 (25.1%) loads strongly on P (+0.92), S (+0.86), Na (+0.76), Zn (+0.73) and Co (+0.73), and moderately on Cd and Cu—the sulfide–Na–P assemblage of the late-stage hydrothermal paragenesis of magnetite–skarns (pyrite/pyrrhotite carrying S, Co, Zn; scapolite/albite carrying Na; apatite carrying P; chalcopyrite/sphalerite carrying Cu, Zn, Cd). RC3 (24.9%) is a carbonate–weathering–redox dimension controlled mainly by physico-chemical properties. Cadmium loads on both RC1 (+0.60) and RC2 (+0.55), reflecting its occurrence in both silicate and sulfide phases; however, this does not affect the central result, which rests on the Co decoupling between the two factors.

The combined evidence indicates that host-rock-derived sulfide-bearing material has been redistributed from the mining complex to the settlement soils [46]: Co is absent from RC1, loads +0.73 on RC2 with the diagnostic sulfide–Na–P assemblage, and the stable site cluster aligns with the waste-dump/open-pit complex. Hierarchical (Ward.D2) and independent k-means clustering resolve the same two-group partition—a “hot” cluster {Konstantinovka, Rudny City} and a “baseline-similar” cluster {background, Pertsevka, Sergeevka} (Table S15), a separation also evident in the PC1–PC2 biplot (Figure S4). In undisturbed terrain, the RC2 signature would be confined to narrow zones over the orebody; its broad expression across four settlements 2.4–4.5 km away, with a cluster matching the source geometry, is consistent with an anthropogenic component superimposed on the regional background (Section 4.1).

### 3.5. Health Risk and Its Uncertainty

**Figure 3:**
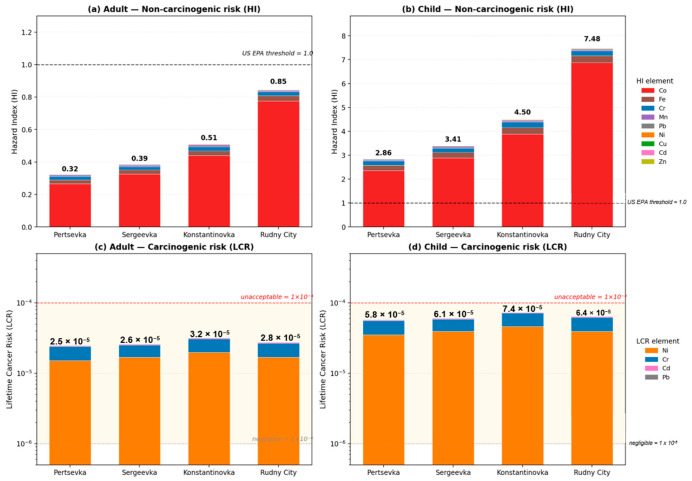
Element-resolved decomposition of US EPA RAGS health risk for the four impacted settlements (*n* = 9 per site; baseline: 100% bioaccessibility, ingestion + dermal + inhalation). (**a**,**b**) Non-carcinogenic hazard index, adult and child; stacked bars decompose HI by element, and dashed line marks HI = 1. (**c**,**d**) Total carcinogenic risk, adult and child (log scale); shaded band = acceptable range 10^−6^–10^−4^, dashed red line = 10^−4^. Cobalt contributes ~88% of the total HI across receptors, while Ni (~62%) and Cr (~34%) dominate TCR.

The third question—which contaminant and pathway?—is answered by the disaggregated hazard index, which identifies cobalt via soil ingestion as the single driver of child hazard. Under the standard US EPA RAGS Part E framework [18] with 100% oral bioaccessibility (Table 3), the mean adult hazard index stays below 1 at all sites (0.32–0.85), though individual samples exceed HI = 1 in 11–33% of cases at Konstantinovka, Sergeevka and Rudny City. For children, HI ≥ 1 occurs in 100% of samples at all four settlements, with site means of 2.86 (Pertsevka), 3.41 (Sergeevka), 4.50 (Konstantinovka) and 7.48 (Rudny City) and a maximum of 11.1 at Rudny City (Figure 3). Decomposition shows cobalt contributing ~88% of the child HI on average (HI_Co = 4.00 of HI_total = 4.56), with Fe (5.7%), Cr (4.2%), Mn (1.0%) and Pb (0.8%) being minor. The total carcinogenic risk for children averages 6.4 × 10^−5^ (range 3.5 × 10^−5^ to 1.03 × 10^−4^), which is within the conventional acceptable range (10^−6^–10^−4^); it is dominated by nickel (62.6% of the mean) and chromium (34.1%), while cobalt, not classified as an oral carcinogen, contributes 0% (full per-element decomposition for both child and adult receptors in Table S16).

**Figure 4:**
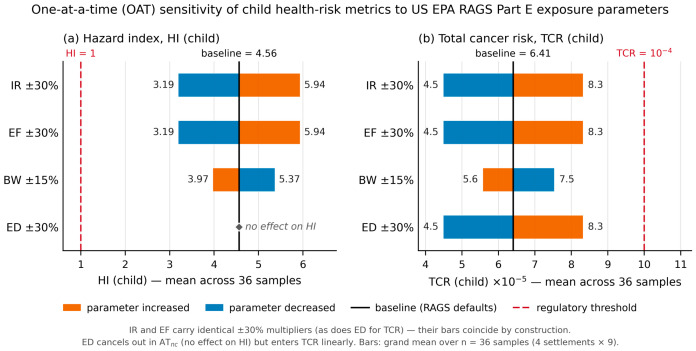
Tornado plot of the one-at-a-time sensitivity of (**a**) child HI and (**b**) child TCR to the US EPA RAGS Part E exposure parameters (*n* = 36; mean across samples), with the stochastic bioaccessibility Monte Carlo percentiles reported in Table 3. Bars show response to + (orange) and − (blue) excursions: IR ±30%, EF ±30%, *BW* ±15% and ED ±30% (ED cancels for HI, affects only TCR). Solid line: baseline (HI = 4.56; TCR = 6.40 × 10^−5^); dashed line in (**a**): HI = 1. The larger cobalt-RfD and bioaccessibility uncertainties are treated in Section 4.4.

Applying literature-based bioaccessibility factors (Co 0.20, Pb 0.50, Cd 0.45, Cr 0.15, Ni 0.20, Cu 0.35, Zn 0.50, Mn 0.30; Fe held at 1.0) to the oral pathway lowers the computed risk but does not remove it (Table 3, final column). The mean child HI remains above one at Rudny City (2.34) and Konstantinovka (1.44) and borderline at Sergeevka (1.10) and Pertsevka (0.93), while the mean child TCR falls to ~1.2 × 10^−5^ at all sites, fully within the acceptable range. The result is robust in both directions of uncertainty. Under one-at-a-time variation of the exposure parameters, the ingestion rate and exposure frequency drive the largest excursions, and child HI ≥ 1 is preserved in every sample under each single-parameter excursion in the baseline scenario; even the joint worst case for the public-health conclusion—the bioaccessibility correction combined with the optimistic exposure excursion—leaves site-mean HI at 0.43–0.71, with 11% of Rudny City samples still ≥ 1 (Figure 4). The 10,000-iteration Monte Carlo on bioaccessibility gives child HI 5th/50th/95th percentiles of 1.82/2.50/4.02 at Rudny City and 1.19/1.58/2.43 at Konstantinovka, so HI > 1 holds even at the 5th percentile—that is, under unfavorable *BAF* assumptions—at these two settlements (per-sample probabilities and per-layer detail in Tables S6 and S7). Because the carcinogenic risk scales linearly with the assumed Cr(VI) fraction, its dependence is analytic and stays within or close to the acceptable range after correction across the full plausible Cr(VI) range (Table S9). These percentiles index sensitivity to the assumed toxicity and bioaccessibility model rather than empirical confidence bounds (Section 4.4).

## 4. Discussion

### 4.1. Why Cobalt: Mineralogy and Mobility of the Cobalt Signal

Cobalt is the only element exceeding the aqua-regia-compatible global baselines in our data, whereas iron—the bulk commodity—stays near the European subsoil baseline despite the 3 Gt orebody. Magnetite–skarn mineralogy explains this: the ore is a poor Co host, whereas the associated late-stage sulfide/sulfarsenide assemblage concentrates it strongly [47] (Co reaching tens of thousands of ppm in sulfarsenides against single-digit ppm in magnetite in the Galinge analogue [11]). Arsenic was not analyzed, so the precise Co host phase cannot be assigned; the PCA association (Co–S–Na–P–Zn on RC2) is most parsimonious with a late-stage sulfide/sulfarsenide source but remains an indirect inference pending direct mineralogy (XRD, SEM-EDS or LA-ICP-MS). When this sulfide-bearing material is disturbed by mining and weathers at the surface, sulfides oxidize far faster than magnetite, releasing Co^2+^ that is soluble and mobile at the neutral-to-slightly-alkaline pH of steppe soils [48,49], whereas dense, magnetite-bound Fe is poorly leached and stays near the source—accounting for the observed near-baseline Fe with strongly elevated, highly mobile Co. The physico-chemical data are consistent with a two-term control on the labile pool—supply and sorption barrier—rather than with a specific dissolution mechanism resolvable on the present design. Mobile Co is inversely related to soil organic matter (ρ = −0.51; ρ = −0.48 with pH partialed out), and a standardized regression of log-transformed mobile Co on pseudo-total Co, humus, pH and exchangeable Ca recovers the same two dominant terms as the companion survey of this complex [46]: Co supply (β_std = +0.45) and the humus sorption barrier (β_std = −0.57), with pH minor (β_std = +0.13). Rudny City combines the highest supply (median pseudo-total Co 154 mg kg^−1^) with the weakest barrier—the lowest humus, exchangeable-base and carbonate contents of the five sites—and accordingly carries by far the largest labile Co pool. The associations of mobile Co with pH, mineralization and carbonate content are, by contrast, single-location contrasts (Section 3.2) and are not interpreted mechanistically here; the geochemical controls on Co lability at this complex are resolved on a larger design in [46], where the same humus-dominated sorption control is identified, and the present data are consistent with that result without adding to it. For the risk assessment, what matters is the size of the labile pool, not the mechanism that sets it.

The high concentration and mobility of Co, its PCA association with the sulfide–Na–P assemblage, and its decoupling from the host-rock matrix (RC1 = −0.10; RC2 = +0.73) are together consistent with release from the late-stage hydrothermal paragenesis of the host skarn, superimposed on an already Co-rich regional background (4× FOREGS at the unimpacted site). Because all three retained factors are mineralogically natural, none represents anthropogenic emissions explicitly, so the case for an anthropogenic contribution rests on spatial expression: a sulfide–Na–P association that would otherwise be confined to narrow zones over the orebody is broadly expressed across four settlements 2.4–4.5 km away, the most affected (Konstantinovka, Rudny City) form a stable cluster matching the waste-dump/open-pit geometry, and the similar background site shows no equivalent expression. The most plausible transport pathway is aeolian: although prevailing S–SSW winds (26.3% of annual hours) carry dust from the settlements towards the mines, episodic reverse N–NNE winds (~24%) transport mine-derived dust back towards them, and the comparatively lower Co at Pertsevka—nearest the tailings facility, where magnetic separation has already removed much of the sulfide fraction—is consistent with this. We treat the anthropogenic contribution as supported but not proven pending direct mineralogical or isotopic (δ^34^S) characterization.

### 4.2. The Paradox of Low Integrated Risk and High Single-Element Risk

**Figure 5:**
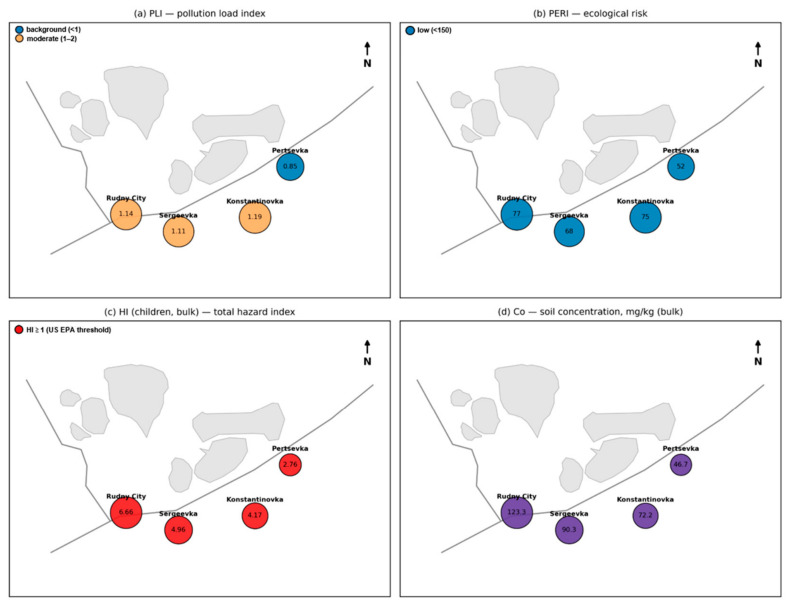
Spatial pattern of four integrated indicators across the four impacted settlements, aggregated over the 0–10 cm profile (*n* = 9 per site). (**a**) Pollution Load Index (PLI; 1 = background, 2 = considerable). (**b**) Potential Ecological Risk Index (PERI; thresholds 150/300/600). (**c**) Child Hazard Index (HI_child, bulk-extractable; threshold HI = 1). (**d**) Site-median bulk Co (mg kg^−1^). The figure captures the central paradox: low ecological-load indices (PLI ≈ 1, PERI well below 150) coexist with child HI > 1 at all four settlements, driven by elevated bulk Co at Rudny City (154 mg kg^−1^).

The most policy-relevant finding is the dissociation between integrated pollution indices and disaggregated health risk metrics (Figure 5). Under the classical framework (*CF*, *I_geo_*, *PLI*, *PERI*) the area is “unpolluted to moderately polluted”: *PERI* reaches at most 90% of the more conservative 110 threshold, no settlement enters the moderate-risk band, and only Co exceeds the Müller [14] *I_geo_* > 1 criterion. This is not an artifact of a generous baseline—the indices use the local background site, itself Co-rich (4.4× FOREGS), so a global baseline would lower them further and widen the gap. The disaggregated picture is the opposite: child HI > 1 is robust to exposure-parameter variation at all four settlements and, at the 5th percentile of literature bioaccessibility, at Rudny City and Konstantinovka. The integrated indices therefore materially understate the child hazard. The contribution is thus less the absolute exceedance than the demonstration that these two frameworks can diverge widely for a single-element, regionally elevated contaminant, governed by toxicity-value and bioaccessibility choices the indices do not encode.

The dissociation has two origins. First, the indices weight elements in ways that need not track toxicological priority: the PERI coefficient for Co (Tr = 5) is six times smaller than for Cd (Tr = 30) [50], even though Co contributes ~88% of the child HI here and Cd < 0.2%. Second, the indices are dominated by abundance ratios to background, so an elevated regional background (Co 35 mg kg^−1^, 4× the European baseline) dampens the CF and Igeo signals at impacted sites. Both effects mean integrated indices under-detect Co-specific risk in iron-skarn regions by construction. Although the 5th-percentile-robust exceedance is confined to Rudny City and Konstantinovka, the exposed population is large: Rudny City alone has 123,240 residents, with ~28,000 minors under 15 and ~10,000 children in the 1–6 bracket of the US EPA child scenario [51]. Because the corrected carcinogenic risk stays within the acceptable range at all sites, the public-health priority is chronic non-carcinogenic exposure to mobile cobalt, not carcinogenic Cr or Ni—a priority echoed by the national standard, whose mobile-form MPC is exceeded only by cobalt.

### 4.3. Implications for Iron Skarn Mining Studies

Most published health risk assessments in mining-impacted soils focus on Cd, Pb and As [1,52,53], reflecting regulatory priorities and the disproportionate study of sulfide-ore districts. Cobalt is rarely listed as a priority, despite its established toxicity (conservative oral RfD 3 × 10^−4^ mg kg^−1^ d^−1^, of the same order as Cd’s 1 × 10^−3^) [5,6,7], its high mobility in oxidized soils and its association with the late-stage sulfide/sulfarsenide paragenesis of magnetite–skarns, a substantial fraction of global iron ore production [54]. The Sokolov–Sarbai case shows that for this widespread deposit class, cobalt may be the principal health-relevant contaminant, eclipsing the conventionally monitored Pb, Cd and As. The limited existing literature supports this: in the Galinge Fe deposit [11], Co occurs in skutterudite, in cobaltite, and as a substituent in arsenopyrite (34,077 ppm Co), with similar assemblages in the Niukutou and Kendekeke skarns [12] and the Han–Xing field [13]; comparable Co-bearing magnetite–skarns occur in Iran (Zanjan district Fe skarns [55]), the Philippines (Larap), the USA (Cornwall, Pennsylvania) [3] and Kazakhstan (Kacharskoye) [19], where waste-material Co is likely to drive similar contamination [56,57], yet health risk assessments are absent.

Detecting this hazard requires mobile-fraction measurement, multivariate source apportionment that decouples Co from the lithogenic factor, and bioaccessibility-corrected risk computation; a study using only integrated indices and pseudo-total concentrations would miss it. The TCR–HI decoupling is informative for risk targeting, because the two metrics probe different element populations: TCR tracks Ni and Cr, which sit at regional-baseline abundance in the settlements, whereas HI tracks Co, the only genuinely enriched element. This is an enrichment-based distinction rather than a strict source assignment, but what matters for the locally manageable component of risk is that Ni and Cr are at background abundance while Co is not. A conventional assessment prioritizing carcinogenic endpoints, as is standard for sulfide-ore districts, would therefore target elements at regional-baseline levels here, whereas the enriched, mobile, locally elevated contaminant is the non-carcinogenic driver cobalt. Cobalt should be added to the standard monitoring suite for magnetite–skarn iron ore operations.

### 4.4. Limitations

Aqua-regia digestion under-recovers silicate-bound Co, so the reported pseudo-total concentrations make the global reference comparisons conservative, and the concentrations were not validated against a certified reference material; the ranked conclusions underlying the public-health interpretation are, however, insensitive to a uniform recovery bias. Arsenic was not analyzed, which limits direct mineralogical identification of the Co-bearing host phases, so the mineralogical attribution rests on the PCA factor structure and on analogy with comparable iron-skarn deposits rather than on direct mineralogical observation. Bioaccessibility was not measured directly: literature-derived BAF values were used, with the Co point estimate (0.20) extrapolated from non-skarn matrices, so direct UBM/SBET measurement (ISO 17924) on the Rudny City and Konstantinovka soils—particularly where the corrected HI is in the borderline 0.9–1.4 range—is a priority for future work. Above all, the absolute hazard is governed by the cobalt oral RfD, a provisional EPA value (PPRTV, not IRIS); a biokinetic re-derivation has since been proposed [58], so the absolute HI carries genuine toxicological uncertainty independent of the exposure parameters, whereas the relative structure (Co dominance, site rank order) is unaffected. Sampling was a single late-summer campaign with limited within-site replication, so seasonal and inter-annual variability is not captured and each settlement is represented by one location; no airborne-dust or water measurements were made, so the aeolian transport pathway is inferred rather than demonstrated. Finally, trace-element transfer into the herbaceous biomass co-located with the soils is the subject of a separate study and is outside the scope of this soil assessment.

## 5. Conclusions

This study asked whether iron ore mining produces a health-relevant soil signal that conventional tools would miss—and it does. Around the Sokolov–Sarbai complex, the classical integrated indices remain low because they average a single-element signal across many elements and reference it to an already elevated background, whereas the disaggregated child hazard index exceeds unity at all four settlements, with cobalt the overwhelming contributor and Rudny City the most affected settlement. After literature-based bioaccessibility correction, the child hazard remains above one at the two most impacted settlements and borderline at the others, while carcinogenic risk stays within the acceptable range, carried by regional-background nickel and chromium rather than a mining increment. The cobalt signal is consistent with selective release from the late-stage sulfide assemblage of the host skarn rather than the magnetite ore, though direct mineralogical confirmation remains for future work. The broader contribution is methodological: for single-element, regionally elevated contaminants, classical integrated indices systematically under-detect a real child health hazard. Therefore, adequate assessment of this widespread site class requires mobile-fraction characterization with bioaccessibility- and reference-dose-explicit risk computation. Site-specific cobalt oral-bioaccessibility measurement and an agreed cobalt reference dose are the priority next steps before the absolute hazard is used for decisions.

## Figures and Tables

**Table 1:** Cobalt concentrations and ratios to pseudo-total (FOREGS, GEMAS) and total (UCC) reference baselines.

Site	Median (mg kg^−1^)	Max (mg kg^−1^)	× FOREGS (Med/Max)	× GEMAS (Med/Max)	× UCC (Med/Max)
Rudny City	154	214	19.2/26.8	19.8/27.6	8.9/12.4
Konstantinovka	71	182	8.9/22.7	9.1/23.3	4.1/10.5
Sergeevka	45	175	5.6/21.9	5.7/22.5	2.6/10.1
Pertsevka	48	70	6.0/8.7	6.2/9.0	2.8/4.0
Background	35	44	4.4/5.5	4.5/5.6	2.0/2.5

**Table 2:** Varimax-rotated factor loadings (bulk-PCA, 16 elements). Bold values indicate factor loadings > 0.5.

Element	RC1 (28.3%)	RC2 (25.1%)	RC3 (24.9%)
Cr	**0.96**	0.12	−0.03
K	**0.86**	0.06	−0.43
Fe	**0.79**	0.23	−0.43
Mg	**0.75**	0.02	**−0.62**
Al	**0.61**	−0.09	**−0.77**
Ni	**0.61**	0.02	**−0.64**
Cd	**0.60**	**0.55**	−0.02
Ca	0.41	0.49	**−0.58**
Pb	0.47	0.06	**−0.52**
Mn	0.34	−0.22	**−0.84**
Cu	0.10	0.44	**−0.82**
Co	−0.10	**0.73**	−0.15
Zn	0.38	**0.73**	−0.17
Na	0.09	**0.76**	**0.53**
S	−0.03	**0.86**	−0.13
P	0.13	**0.92**	0.00

**Table 3:** Health risk assessment summary (baseline scenario, 100% bioaccessibility). The child hazard index is computed under the US EPA PPRTV cobalt reference dose (3 × 10^−4^ mg kg^−1^ d^−1^).

Site	HI Adult Mean (Max)	HI Child Mean (Max)	% HI (Child) ≥ 1	TCR Child Mean (Max)	HI Child, BAF-Corrected Mean
Rudny City	0.85 (1.26)	7.48 (11.11)	100	6.4 × 10^−5^ (1.03 × 10^−4^)	2.34
Konstantinovka	0.51 (1.08)	4.50 (9.55)	100	7.4 × 10^−5^ (9.6 × 10^−5^)	1.44
Sergeevka	0.39 (1.06)	3.41 (9.32)	100	6.1 × 10^−5^ (1.02 × 10^−4^)	1.10
Pertsevka	0.32 (0.45)	2.86 (3.92)	100	5.8 × 10^−5^ (8.0 × 10^−5^)	0.93

## Data Availability

The data presented in this study are available within the article and its Supplementary Materials.

## Supplementary Materials

The following supporting information can be downloaded at https://www.mdpi.com/article/10.3390/toxics14070617/s1. Figure S1: Vertical depth profiles of eight priority trace metals across five sites; Figure S2: Site-level distribution of mobile (NH_4_OAc, pH 7) fractions of eight priority trace metals; Figure S3: Spearman correlation heatmap, mobile-element concentrations vs soil physico-chemical properties; Figure S4: PCA biplot of soil samples in PC1–PC2 space; Table S1: Raw per-sample soil data (*n* = 45). Table S2: ICP-OES instrument conditions and analytical performance; Table S3: Soil physico-chemical properties; Table S4: PCA varimax-rotated loadings, variance explained and communalities (full 22-variable model); Table S5: Risk-model input parameters; Table S6: Sensitivity of child HI and TCR to RAGS exposure parameters; Table S7: Bioaccessibility-corrected HI and TCR; Table S8: Monte-Carlo HI (stochastic BAF, 10,000 iterations); Table S9: Cr(VI)-fraction sensitivity of carcinogenic risk; Table S10: Statistical tests; Table S11: Spearman correlations, mobile-element concentrations vs. soil physico-chemical properties; Table S12: Descriptive statistics, pseudo-total and mobile concentrations (16 elements); Table S13: Trace-element ratios to reference baselines; Table S14: Integrated pollution indices; Table S15: Cluster analysis (HCA Ward.D2 and k-means); Table S16: Per-element health-risk decomposition.
