# Supplementary material for: Hidden Cobalt Hazards in Children: A Soil Risk Assessment at the Sokolov–Sarbai Complex
Source: Toxics. 2026 Jul 15;14(7):617. doi: 10.3390/toxics14070617 (PMC13416588; doi:10.3390/toxics14070617)
Supplement: Supplementary file 1 [file toxics-14-00617-s001.zip › toxics-4386014-Supplementary Figures.pdf]

Supplementary Materials

Hidden Cobalt Hazards in Children: A Soil Risk Assessment at the Sokolov–Sarbai Complex

Bekmyrza Zh., Yskak A., Paramonova T.A., Irzhanov Zh.B. et al. • Toxics 2026, 14, x.

This document contains Supplementary Figures S1–S4 and the list of Supplementary Tables. The complete set of Supplementary Tables (Tables S1–S16) is provided as a separate Microsoft Excel workbook, Supplementary_Tables.xlsx, with one table per worksheet (tabs named Tbl_S1 … Tbl_S16). Tables consolidated from several analyses carry their constituent parts as labelled sub-blocks within a single worksheet.

List of Supplementary Materials

Supplementary Figures (this file)

Figure S1: Vertical depth profiles of eight priority trace metals across five sites (Section 3.1).

Figure S2: Site-level distribution of mobile (NH_4OAc, pH 7) fractions of eight priority trace metals (Section 3.2).

Figure S3: Spearman correlation heatmap: mobile-element concentrations vs soil physico-chemical properties (Section 3.2).

Figure S4: PCA biplot of soil samples in PC1–PC2 space (Section 3.4).

Supplementary Tables (in Supplementary_Tables.xlsx)

Table S1: Raw per-sample soil data (n = 45).

Table S2: ICP-OES instrument conditions and analytical performance.

Table S3: Soil physico-chemical properties.

Table S4: PCA varimax-rotated loadings, variance explained and communalities (full 22-variable model).

Table S5: Risk-model input parameters.

Table S6: Sensitivity of child HI and TCR to RAGS exposure parameters.

Table S7: Bioaccessibility-corrected HI and TCR.

Table S8: Monte-Carlo HI (stochastic BAF, 10,000 iterations).

Table S9: Cr(VI)-fraction sensitivity of carcinogenic risk.

Table S10: Statistical tests.

Table S11: Spearman correlations, mobile-element concentrations vs. soil physico-chemical properties.

Table S12: Descriptive statistics, pseudo-total and mobile concentrations (16 elements).

Table S13: Trace-element ratios to reference baselines.

Table S14: Integrated pollution indices.

Table S15: Cluster analysis (HCA Ward.D2 and k-means).

Table S16: Per-element health-risk decomposition.

Supplementary Figures

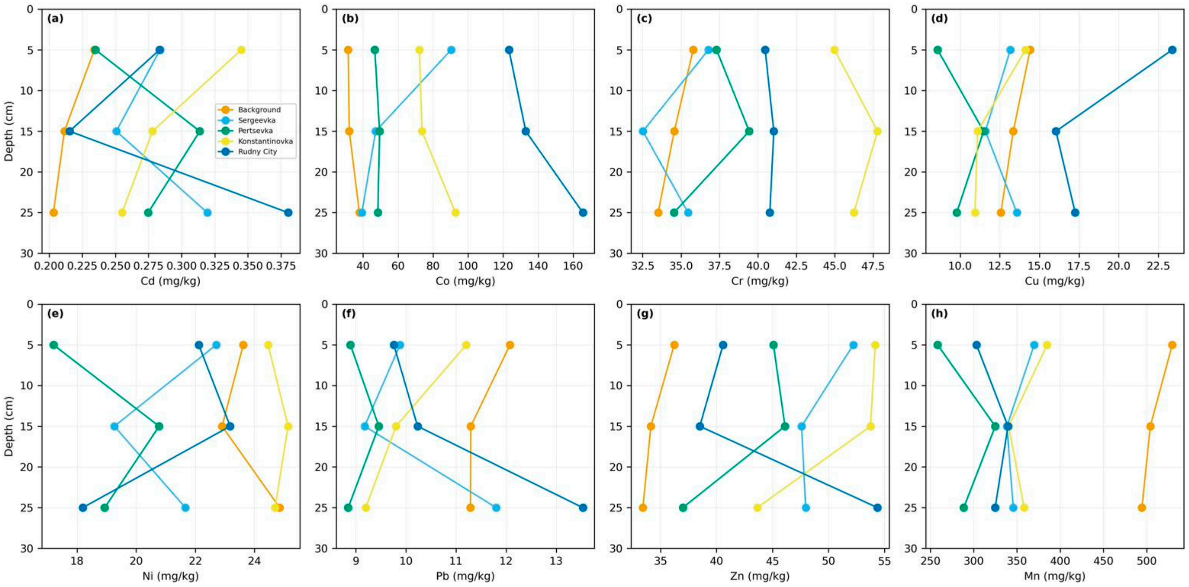

Figure S1. Vertical depth profiles of eight priority trace metals (mean concentration in mg kg^{-1} dry weight) in the 0–10 / 10–20 / 20–30 cm horizons across five sites. Panels: (a) Cd, (b) Co, (c) Cr, (d) Cu, (e) Ni, (f) Pb, (g) Zn, (h) Mn. Sites are colour-coded as in Figure 2. Panel (b) shows a moderate increase of Co with depth at Rudny City (130 → 165 mg kg^{-1} from 5 to 25 cm); the profiles are reported descriptively and are not used for source attribution (Section 3.1).

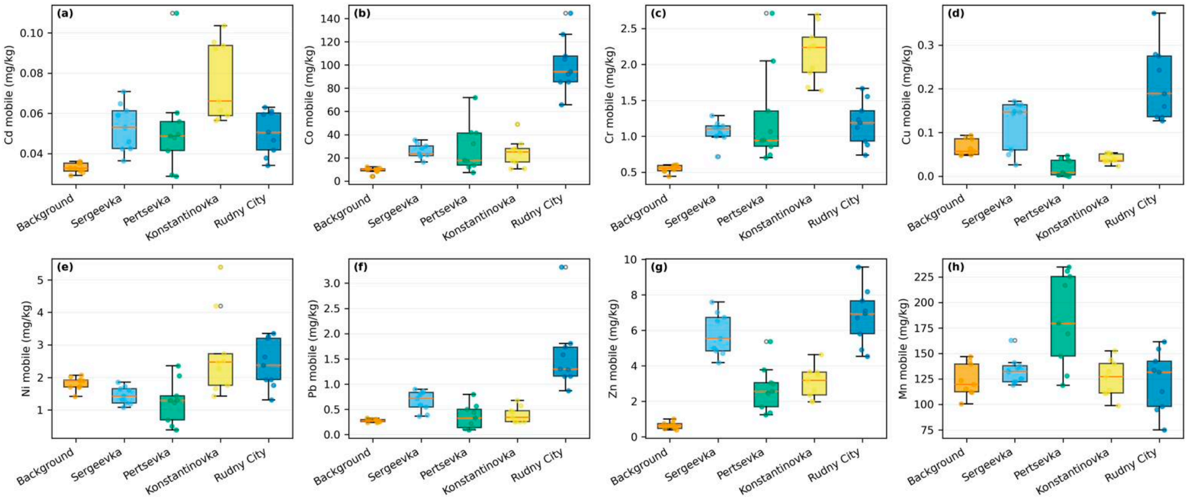

Figure S2. Site-level distribution of mobile (neutral 1 M ammonium acetate, pH 7) fractions of the same eight priority trace metals as in Figure 2, in mg kg^{-1} dry weight. Panels (a–h): Cd, Co, Cr, Cu, Ni, Pb, Zn, Mn. Note the order-of-magnitude contrast between cobalt at Rudny City (median ≈ 95 mg kg^{-1}) and the other sites, including the background (≤ 30 mg kg^{-1}), which is the strongest mobility signal across all elements measured. Site abbreviations and sample design as in Figure 2.

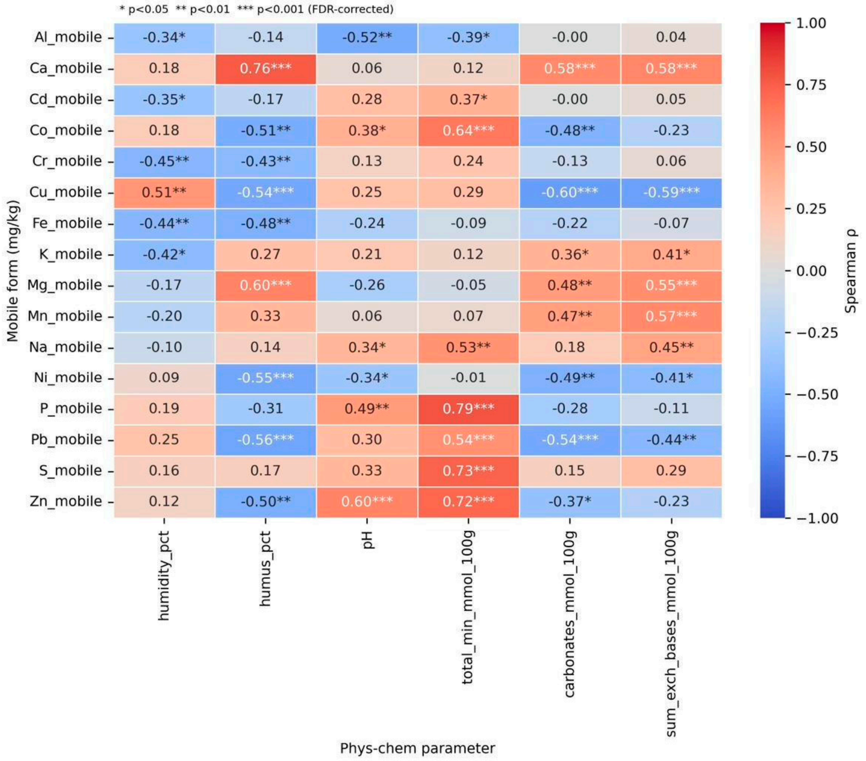

Figure S3. Spearman rank-correlation heatmap between mobile-fraction (neutral 1 M ammonium acetate, pH 7) concentrations of 16 elements (rows) and six soil physico-chemical properties (columns): hygroscopic humidity, humus, pH, total mineralisation, exchangeable carbonates and sum of exchangeable bases. Cell values are Spearman's ϱ; significance after Benjamini–Hochberg false-discovery-rate correction is indicated by * $p < 0.05$, ** $p < 0.01$, *** $p < 0.001$. n = 45 (all five sites). Of the properties examined, soil organic matter (humus) is the only one whose association with mobile cobalt is robust across the dataset: the correlation is negative (ϱ = −0.51), survives control for pH (partial ϱ = −0.48), and retains its sign and significance within the impacted settlements alone (ϱ = −0.47, n = 36) and under leave-one-site-out resampling. The apparent correlations of mobile Co with pH, total mineralisation and exchangeable carbonates are between-site contrasts carried by single locations — they collapse when the leverage site is removed (the pH association falls to ϱ = −0.03 with the background site excluded, and the carbonate association to ϱ = +0.01 with Rudny City excluded) — and are therefore not interpreted as mechanistic controls here (Section 4.1).

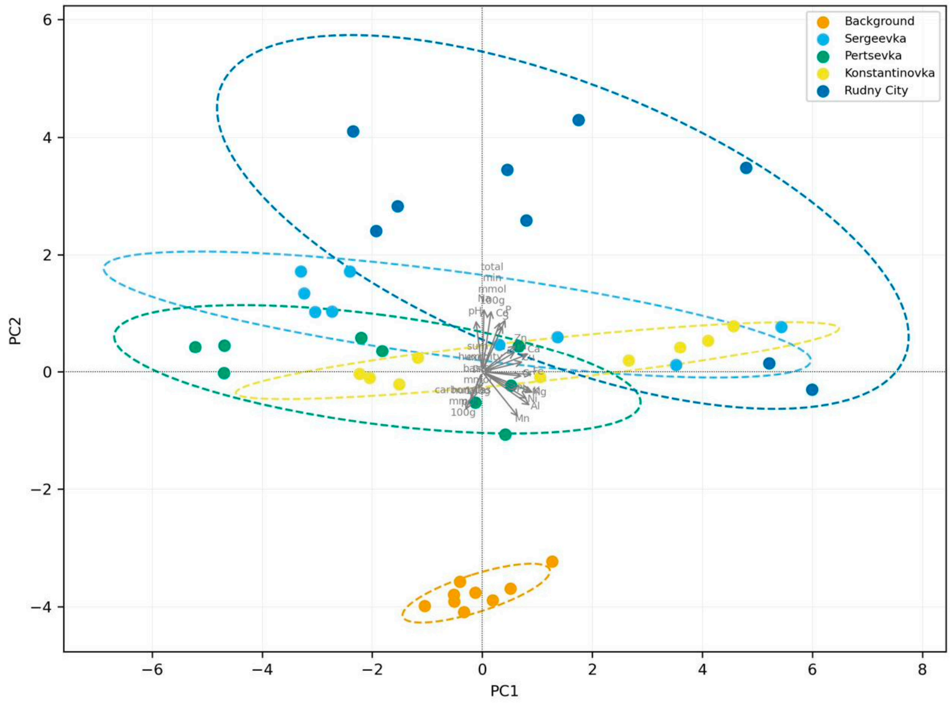

Figure S4. Principal component analysis (PCA) biplot of soil samples in the PC1–PC2 space. Variables: 16 bulk-extractable elements and six physico-chemical properties (standardised to z-scores; varimax-rotated component loadings are reported in Table 2 and Table S3). Samples are coloured by site and grouped by 95%-confidence ellipses. Loadings are shown as grey vectors. PC1 (47.1% of variance) separates the carbonate-rich, mineralisation-loaded Rudny City profiles from the background; PC2 (22.9%) isolates the cobalt axis on which the Rudny City samples cluster.

Supplementary Notes

Full-model PCA (source attribution). The full-model PCA (22 variables including six physico-chemical predictors) confirms the same factor structure as the bulk model but redistributes part of the Co variance between RC2 (sulfide–Na–P) and RC3 (here an inverse-carbonate factor including total mineralisation and pH; Table S3). Co loadings in the full model are RC1 = +0.05, RC2 = +0.50 and RC3 = −0.60. Communalities are h^2_Co = 0.56 (bulk) and 0.60 (full), within the acceptable range for source-apportionment interpretation. Both models converge on the central finding: cobalt is completely decoupled from the lithogenic factor RC1 and is the signature element of the sulfide–Na–P assemblage.

Cluster analysis and source geometry. Hierarchical cluster analysis on site-mean profiles (Ward.D2; Table S14) and independent k-means clustering on the same data both yield identical site groupings for k = 2: a 'hot' cluster comprising Konstantinovka and Rudny City, and a 'baseline-similar' cluster comprising Pertsevka, Sergeevka and the background site. This stable two-way partition is structurally consistent with the spatial distribution of source bodies (Figure 1): both 'hot' cluster settlements lie adjacent to and downwind from the open-pit and waste-dump complex along the prevailing wind axis, with Rudny City ~4.5 km south-southwest of Open-pit OP1 and Konstantinovka ~4.4 km south of Waste-dump WD2.1; both positions lie within episodic reverse-direction (N–NNE) wind sectors that occur ~24% of the time and carry mine-derived dust towards the settlements. Pertsevka, the settlement most distant from the open-pit/waste-dump complex and primarily exposed to the tailings storage facility (whose material is depleted in sulfides through magnetic separation of the ore), shows the smallest Co signal.
